# Up and down waves of glycemic control and lower-extremity amputation in diabetes

**DOI:** 10.1186/s12933-021-01325-3

**Published:** 2021-07-06

**Authors:** Paola Caruso, Lorenzo Scappaticcio, Maria Ida Maiorino, Katherine Esposito, Dario Giugliano

**Affiliations:** 1 Division of Endocrinology and Metabolic Diseases, Department of Advanced Medical and Surgical Sciences, University of Campania Luigi Vanvitelli, Naples, Italy; 2 Ph.D. of Translational Medicine, Chair of Endocrinology and Metabolic Diseases, Department of Advanced Medical and Surgical Sciences, University of Campania Luigi Vanvitelli, Naples, Italy; 3 Diabetes Unit, Department of Advanced Medical and Surgical Sciences, University of Campania Luigi Vanvitelli, Naples, Italy

## Abstract

Lower extremity amputations (LEA) are associated with a high mortality and medical expenditure. Diabetes accounts for 45% to 70% of LEA and is one of the most potent risk factors for peripheral artery diseases (PAD). The existence of a link between the recent relaxation of glycemic targets and the resurgence of LEA is suggested from the analysis of adult participants in the National Health and Nutrition Examination Survey (NHANES) between 2010 and 2015, when diabetes-related LEA increased by more than 25% associated with a decline in glycemic control. Indeed, in “the perfect wave” of NHANES, including the years 2007–2010, there was the highest number of diabetic people with hemoglobin A1c (HbA1c), non-high-density lipoprotein (HDL) cholesterol and blood pressure levels at their respective targets, associated with the lowest number of LEA. Until now, the ACCORD study, testing the role of aggressive vs conventional glucose control, and the LEADER trial, evaluating the effects of liraglutide versus placebo, have shown a reduced incidence of LEA in people with type 2 diabetes. The results of ongoing clinical trials involving glucagon-like peptide-1 receptor agonists (GLP-1RA, liraglutide or semaglutide) hopefully will tell us whether the wider use of these drugs may provide additional vascular benefits for diabetic people affected by PAD to decrease their risk of LEA.

More adults with diabetes in the US have suboptimal glycemic control now compared to 10 years ago [1]. After more than a decade of progress, glycemic control declined in adult NHANES (National Health and Nutrition Examination Survey) participants with diagnosed diabetes. In parallel with the reduced focus on meticulous blood glucose control, a resurgence in vascular diabetic complications is under way [2]. During the early 1990s, patients with diabetes had an increased risk of lower-extremity amputation (LEA) as compared with people without diabetes, with an incidence of 58 versus only 3 cases/10.000 persons/year, respectively [3]. Later, this risk difference declined sharply by ≈ 40% between 2000 and 2009, especially through improvements in diabetes care [3]. However, in only five years, between 2010 and 2015, an increase in diabetes-related LEA has frustrated more than a third of the positive results achieved in the past decade [4]. LEA increased by more than 25%, with a rebound in rates most apparent in young (aged 18–44 years) and middle-aged adults (aged 45–64 years), as compared with older adults, in whom the long-term reductions in rates have reached a plateau. The suspicion about a link between the relaxation of glycemic targets and the resurgence of LEAs is strong. Figure 1 shows the results of the NANHES waves in the first two decades of this century. We have titled the Perfect Wave the one between 2007–2010, in which there was the highest number of diabetic people with hemoglobin A1c (HbA1c), non-high-density lipoprotein (HDL cholesterol) and blood pressure levels at their respective targets, associated with the lowest number of LEA. The following waves were characterized by the relaxation of targets associated with increasing number of LEA.

**Fig. 1:**
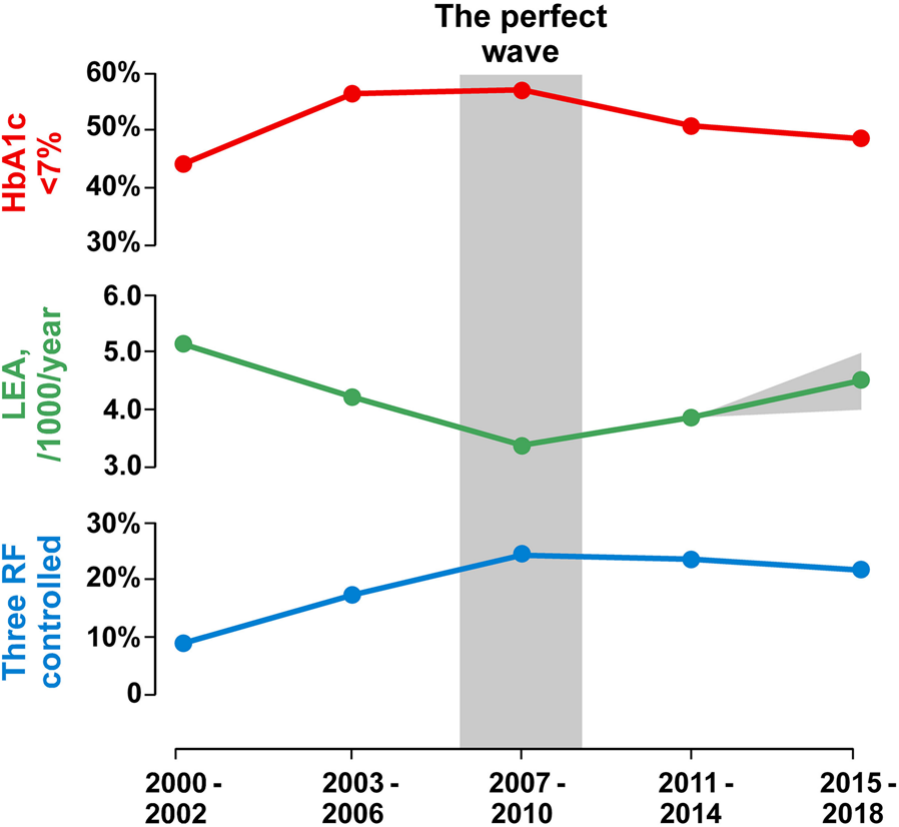
Overview of the five NHANES waves (2000 to 2018) that assessed the prevalence of optimal glycemic control (HbA1c < 7%, red circles), incidence of LEA (lower-extremity amputations, number/1000/year, green circles) and prevalence of three risk factor (RF) control (HbA1c < 7%, non-HDL cholesterol < 130 mg/dl, blood pressure < 140/90 mm Hg, blue circles) in participants with diagnosed diabetes. The name perfect wave has been given to the central wave (2007–2010) because it was associated with the highest prevalence of participants in whom all three targets were simultaneously achieved, and the lowest incidence of LEA. The gray triangle at the end of the green curve indicates the possible scenario of LEA, as only data related to year 2015 are available

## The burden of LEA

Peripheral artery disease (PAD) is a major cause of LEA, which is burdened with a high mortality and medical expenditure [5]. Of the ≈ 150,000 non-traumatic LEA that occur every year in the USA, most cases occur in patients with diabetes, which accounts for 45 to 70% of LEA; moreover, diabetes is one of the most potent risk factors for PAD. There are other factors that in addition to or independent of PAD may cause or contribute to LEA in patients with diabetes, including microvascular abnormalities with critical limb ischemia, and the diabetic foot associated with a series of neuropathic abnormalities [6]. The presence and severity of microvascular complications contribute independently to increase the risk of mortality and cardiovascular events. Microangiopathy and macroangiopathy are considered a continuum of systemic vascular damage [6]. Although patients with diabetes and PAD are among those at the highest risk for LEA, amputation is often preventable, even among patients with advanced PAD.

## The glucose hypothesis

The glucose hypothesis claims that long-lasting hyperglycemia plays an important role in the increased frequency of vascular complications that occur in people with diabetes, including micro and macrovascular complications. The three major trials [7–9], built up in the first decade of this century to verify the glucose hypothesis, have shown that intensive glycemic control had no cardiovascular benefit and increased the risk of hypoglycemia. A later meta-analysis [10], that also included the older UKPDS (United Kingdom Prospective Diabetes Study) trial, confirmed a modest reduction (9%) in major macrovascular events with aggressive glucose lowering, associated with two times and half more major hypoglycemic events. These findings renewed support for individualized glycemic targets, especially for prevention of hypoglycemia for older adults. An unintended consequence of this recommendation by scientific and clinical organizations may have been the relaxation of glycemic control targets for younger adults.

## The glucose hypothesis for LEA

In epidemiologic studies, there is a dose-dependent effect of hyperglycemia on increased risk of PAD: every 1% increase in HbA1c is associated with a 26% increased risk of PAD [11]. In contrast to other cardiovascular outcomes, better glucose control seems associated to lower risk of LEA in patients with diabetes. The ACCORD (Action to Control Cardiovascular Risk Factors in Diabetes) trial was one of the three controlled clinical trials designed to verify the glucose hypothesis in type 2 diabetes by comparing the effect of intensive glycemic control (HbA1c target < 6.0%) versus standard glycemic control (HbA1c target: 7.0–7.9%). While the glycemic arm of the trial was discontinued prematurely because the aggressive lowering of glycemia was associated with increased mortality [12], the participants were all transitioned to standard glycemia management and were followed for additional 17 months, until completion of the blood pressure and lipid arms. A secondary analysis from the ACCORD trial [13] has reported that the hazard ratio of LEA was 31% lower in those diabetic patients primarily allocated to intensive glucose control group versus those allocated to the standard glycemic control, and the benefit was maintained over more than 7-year follow-up period. Moreover, HbA1c was the strongest predictor of LEA, regardless of original group assignment.

Until now, the results of the extended ACCORD trial are alone to support a benefit of intensive glucose control on the risk of LEA in type 2 diabetes. Data of the other trials of intensive glucose control related to amputation do not appear to have been published.

## The novel hypothesis

The recent paradigm shift in diabetes therapy [14] claims that the adoption of newer drugs, such as glucagon-like peptide-1 receptor agonists (GLP-1RA) and sodium-glucose cotrasporter-2 (SGLT-2) inhibitors, for the management of type 2 diabetes may improve the poor cardiovascular outlook of the diabetic patient. In the LEADER (Liraglutide Effect and Action in Diabetes: Evaluation of Cardiovascular Outcome Results) trial [15], the GLP-1RA liraglutide was associated with a 35% lower risk of diabetic foot ulceration–related amputations compared with placebo, with no differences in foot infections, involvement of underlying structures, or peripheral revascularization in the main analysis. Data of the other trials with different GLP-1RA, including lixisenatide, exanatide, semaglutide, dulaglutide and albiglutide, related to amputation do not appear to have been published.

In contrast with a putative beneficial effect of liraglutide, an observational study of about one million US patients with diabetes has reported a higher risk (≈ 2 times higher) of leg amputations among new users of SGLT-2 inhibitors compared with users of other antidiabetic medications [16]. However, increased risk of amputation has not been reported in clinical trials of SGLT-2 inhibitors such as empagliflozin, dapagliflozin, ertugliflozin and sotagliflozin [17].

According to the adage that a swallow does not make spring, the association between the use of liraglutide and reduction in amputation in diabetic patients at high cardiovascular risk could be due to chance, but merits further investigation.

There are at least three ongoing trials evaluating the effect of GLP-1RA on some surrogate endpoints in diabetic patients affected by PAD. Two trials (NCT04146155, NCT04881110) will evaluate the efficacy of a six-month treatment with liraglutide of pain-free walking distance or peripheral transcutaneous oxygen tension. Another trial (NCT04560998) will assess the effect of a 52-week treatment with semaglutide on maximum walking distance.

## Conclusions

PAD is strictly related to the onset and the prognosis of diabetic foot ulcers, determining an increase of the risk of LEA [18]. In the World Health Organization mortality database collecting data in 108 countries from 2000 to 2016, a proportion of 27.1% deaths (515.293 cases) was referred to macrovascular complications affecting the peripheral arteries [19]. The US national decline in glycemic control after 2010 has been associated with a resurgence of LEA in the young and middle-aged US diabetic population. There is an urgent need for interventions and strategies that safely resume progress in diabetes risk-factor control (glucose, cholesterol, and blood pressure). The results of ongoing clinical trials will tell us whether the wider use of some GLP-1RA (primarily liraglutide and hopefully semaglutide) may help the 200 million people worldwide affected by PAD [20] to decrease their risk of LEA.

## Data Availability

All data generated or analyzed during this study are included in this published article.
